# Transcripts of two *ent*-copalyl diphosphate synthase genes differentially localize in rice plants according to their distinct biological roles

**DOI:** 10.1093/jxb/eru424

**Published:** 2014-10-21

**Authors:** Tomonobu Toyomasu, Masami Usui, Chizu Sugawara, Yuri Kanno, Arisa Sakai, Hirokazu Takahashi, Mikio Nakazono, Masaharu Kuroda, Koji Miyamoto, Yu Morimoto, Wataru Mitsuhashi, Kazunori Okada, Shinjiro Yamaguchi, Hisakazu Yamane

**Affiliations:** ^1^Department of Bioresource Engineering, Yamagata University, Yamagata 997-8555, Japan; ^2^Graduate School of Bioagricultural Sciences, Nagoya University, Nagoya 464-8601, Japan; ^3^Rice Physiological Research Team, NARO Agricultural Research Center, Niigata 943-0193, Japan; ^4^Biotechnology Research Center, The University of Tokyo, Tokyo 113-8657, Japan; ^5^Laboratory of Bioactive Molecules, Graduate School of Life Sciences, Tohoku University, Sendai 980-8577, Japan

**Keywords:** Biosynthetic enzyme, diterpene, gene expression, gibberellin, growth, rice.

## Abstract

Gibberellins (GAs) are diterpenoid phytohormones that regulate various aspects of plant growth. Tetracyclic hydrocarbon *ent*-kaurene is a biosynthetic intermediate of GAs, and is converted from geranylgeranyl diphosphate, a common precursor of diterpenoids, via *ent*-copalyl diphosphate (*ent*-CDP) through successive cyclization reactions catalysed by two distinct diterpene synthases, *ent*-CDP synthase and *ent*-kaurene synthase. Rice (*Oryza sativa* L.) has two *ent*-CDP synthase genes, *OsCPS1* and *OsCPS2*. It has been thought that *OsCPS1* participates in GA biosynthesis, while *OsCPS2* participates in the biosynthesis of phytoalexins, phytocassanes A–E, and oryzalexins A–F. It has been shown previously that loss-of-function *OsCPS1* mutants display a severe dwarf phenotype caused by GA deficiency despite possessing another *ent*-CDP synthase gene, *OsCPS2*. Here, experiments were performed to account for the non-redundant biological function of *OsCPS1* and *OsCPS2*. Quantitative reverse transcription–PCR (qRT–PCR) analysis showed that *OsCPS2* transcript levels were drastically lower than those of *OsCPS1* in the basal parts, including the meristem of the second-leaf sheaths of rice seedlings. qRT–PCR results using tissue samples prepared by laser microdissection suggested that *OsCPS1* transcripts mainly localized in vascular bundle tissues, similar to *Arabidopsis CPS*, which is responsible for GA biosynthesis, whereas *OsCPS2* transcripts mainly localized in epidermal cells that address environmental stressors such as pathogen attack. Furthermore, the *OsCPS2* transgene under regulation of the *OsCPS1* promoter complemented the dwarf phenotype of an *OsCPS1* mutant, *oscps1-1*. The results indicate that transcripts of the two *ent*-CDP synthase genes differentially localize in rice plants according to their distinct biological roles, *OsCPS1* for growth and *OsCPS2* for defence.

## Highlight

Expression of the diterpene synthase gene for gibberellin biosynthesis occurs in tissues different from those in which its isoform for phytoalexin biosynthesis is expressed, reflecting their distinct biological roles in rice.

## Introduction

**Fig. 1.**
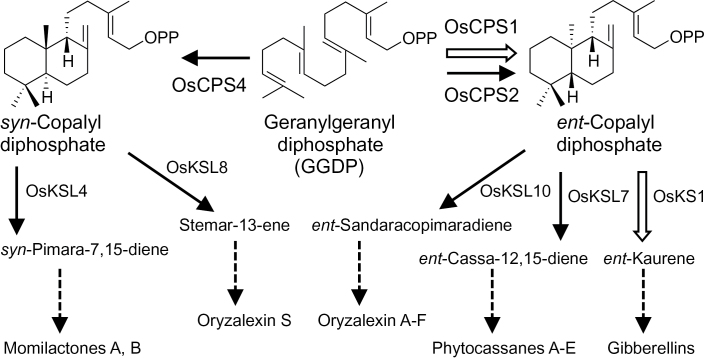
Early steps in the biosynthetic pathway of labdane-related diterpenoids in rice. The steps in the formation of intermediate cyclic hydrocarbons of gibberellins (open arrows) and diterpene phytoalexins (solid arrows) are shown with the names of diterpene cyclases next to the arrows.

Diterpenoids are a class of terpenoids mainly derived from the C20 prenyl substrate geranylgeranyl diphosphate (GGDP). The linear substrate GGDP is converted into various cyclic hydrocarbons by specific diterpene cyclases. The carbon skeletons are successively chemically modified by enzymes including P450 monooxygenases, dehydrogenases, methyltransferases, glucosyl transferases, and others. Gibberellins (GAs) are labdane-related diterpene phytohormones that regulate various aspects of plant growth, such as germination, stem elongation, and flowering (Yamaguchi, 2008; Hedden and Thomas, 2012). GAs are biosynthesized from the intermediate tetracyclic hydrocarbon *ent*-kaurene, which is converted from GGDP by two-step cyclization (Fig. 1). The two steps are catalysed by two distinct diterpene cyclases, *ent*-copalyl diphosphate (*ent*-CDP) synthase and *ent*-kaurene synthase. Other GA biosynthetic genes have been identified and characterized in detail (Yamaguchi, 2008; Hedden and Thomas, 2012).

Rice (*Oryza sativa* L.) produces not only GAs but also labdane-related diterpene phytoalexins, including phytocasssanes (Koga *et al.*, 1995, 1997; Yajima and Mori, 2000), oryzalexins (Akatsuka *et al.*, 1985; Kato *et al*., 1993, 1994; Tamogami and Mitani, 1993), and momilactones (Kato *et al.*, 1973; Cartwright *et al.*, 1981). Phytoalexins are low molecular weight compounds produced for defence against pathogens (Ahuja *et al.*, 2012). The biosynthetic hydrocarbon intermediates of phytocassanes A–E, oryzalexins A–F, oryzalexin S, and momilactones A and B are *ent*-cassa-12, 15-diene, *ent*-sandaracopimaradiene, stemar-13-ene, and *syn*-pimara-7, 15-diene, respectively (Fig. 1). The first and last two are converted from GGDP through *ent*-CDP and *syn*-CDP, a diastereomer of *ent*-CDP, respectively (Fig. 1). The diterpene cyclase genes responsible for these steps have been identified in the rice genome (Peters, 2006; Toyomasu, 2008): *OsCPS2*, *OsCPS4*, *OsKSL4*, *OsKSL7*, *OsKSL8*, and *OsKSL10* (Fig. 1). Diterpene cyclases responsible for *ent*-kaurene biosynthesis in rice are OsCPS1 and OsKS1 (Fig. 1) because *OsCPS1* and *OsKS1* loss-of-function mutants display a severe dwarf phenotype caused by GA deficiency (Sakamoto *et al.*, 2004). Thus, rice has two *ent*-CDP synthase genes, *OsCPS1* for GA biosynthesis and *OsCPS2* for phytoalexin biosynthesis. Although rice possesses another *ent*-CDP synthase gene, *OsCPS2*, loss-of-function *OsCPS1* mutants display a severe dwarf phenotype (Sakamoto *et al.*, 2004). This finding indicates that *OsCPS2* cannot prevent the *OsCPS1* mutant dwarf phenotype and has no redundant biological function with *OsCPS1*. *OsCPS2* expression is up-regulated, unlike *OsCPS1*, in response to biotic or abiotic stress, including UV irradiation, chitin elicitor treatment, jasmonic acid treatment, and pathogen attack (Otomo *et al.*, 2004; Prisic *et al.*, 2004; Sakamoto *et al.*, 2004; Okada *et al.*, 2007; Hasegawa *et al.*, 2010). Thus, the positive correlation of *OsCPS2* expression and phytoalexin accumulation strongly suggested that *OsCPS2* is responsible for phytoalexin biosynthesis.

A difference in enzymatic properties between recombinant OsCPS1 and OsCPS2 was previously shown (Hayashi *et al.*, 2008). However, these studies did not clearly explain the non-redundant function of *OsCPS1* and *OsCPS2*. Therefore, herein the localization of these transcripts in rice plants and the subcellular localization of their translated products were compared in order to account for their non-redundancy. Consequently, it was found that transcripts of *OsCPS1* and *OsCPS2* are differentially localized in rice plants according to their biological roles, and a complementation experiment using an *OsCPS1* mutant by ectopic expression of *OsCPS2* was performed to verify the conclusion genetically.

## Materials and methods

### Plant materials

Rice (*Oryza sativa* L. cv. Nipponbare) seedlings were grown at 25 °C under continuous light conditions in a growth chamber until the third-leaf stage, and the upper and basal 2cm regions were excised from the second-leaf sheath and used for quantitative analyses of transcripts. The *oscps1-1* mutant (Sakamoto *et al.*, 2004) is a *Tos17*-inserted mutant NE3024 (Nipponbare background; Supplementary Fig. S1 available at *JXB* online), and its M_1_ seeds were purchased from the Rice Genome Resource Center, National Institute of Agrobiological Sciences. A heterozygous M_1_ plant was used for transformation.

### Laser microdissection

The upper parts of the second-leaf sheaths of third-leaf stage rice seedlings were fixed in ethanol:acetic acid (3:1, v/v). Paraffin embedding and laser microdissection were performed as previously described (Takahashi *et al.*, 2010). In brief, 16 μm thin sections were cut from paraffin blocks and mounted on PEN membrane class slides (Life Technologies Corporation, CA, USA) for laser microdissection. The vascular bundle-rich and epidermis-rich tissues were collected from the leaf sheath sections using a Veritas Laser Microdissection System LCC1704 (Life Technologies Corporation). In addition, mesophyll-rich tissues were collected.

### RNA extraction and quantitative reverse transcription–PCR (qRT–PCR)

Total RNA was extracted and purified from the leaf sheath samples using an RNAqueous kit (Invitrogen, Carlsbad, CA, USA), and cDNA templates were synthesized from total RNA using a QuantiTect Reverse Transcription kit (Qiagen KK, Tokyo, Japan). Total RNA was extracted from tissue samples prepared by laser microdissection, using a PicoPure RNA Isolation kit (Life Technologies Corporation) according to the manufacturer’s instructions. The quantity of RNA was determined by the Quant-iT RiboGreen RNA Assay Kit (Life Technologies Corporation). RNA integrity was assessed using an Agilent 2100 Bioanalyzer with an Agilent RNA 6000 Nano kit (Agilent Technologies, CA, USA). cDNA templates were synthesized and amplified using a WT-Ovation RNA Amplification System version 1.0 (NuGEN Technologies, CA, USA). qRT–PCR was performed using a Thermal Cycler Dice Real Time System TP800 (Takara Bio, Otsu, Japan) and SYBR Premix Ex Taq Perfect Real Time version 2 (Takara Bio), as previously described (Toyomasu *et al.*, 2008). The concentration of each transcript was normalized to 18S rRNA. Nucleotide sequences of the primers used are listed in Supplementary Table S1 at *JXB* online. The primer set for *OsCPS1* can amplify a fragment derived from the wild-type *OsCPS1* mRNA but not a fragment from *Tos17*-inserted *OsCPS1* mRNA (Supplementary Fig. S1).

### Green fluorescent protein (GFP) assay

The cDNA fragments encoding the N-terminal 153 and 108 amino acids of OsCPS1 and OsCPS2, respectively (OsCPS1-N153 and OsCPS2-N108, Supplementary Fig. S2A at *JXB* online), were amplified by RT–PCR using the Advantage 2 PCR System (Takara Bio) and subcloned into the pGEM-T Easy vector (Promega, WI, USA). Primer sets (sense/antisense) for amplification of OsCPS1-N153 and OsCPS2-N108 fragments are XbaI-CPS1-F/BamHI-CPS1-N153-R and XbaI-CPS2-F/BamHI-CPS2-N108-R, respectively (Supplementary Table S2). A fragment encoding the chimeric peptide OsCPS1-N91:OsCPS2-N108 (Supplementary Fig. S2B) was prepared by fusion of two corresponding cDNA fragments. Fragments encoding the N-terminal 91 amino acids of OsCPS1 (OsCPS1-N91) and OsCPS2-N108 were amplified using KOD Plus version 2 (Toyobo, Osaka, Japan) and the primer sets XbaI-CPS1-F/CPS1N91CPS2-R and CPS1N91CPS2-F/BamHI-CPS2-N108-R, respectively (Supplementary Table S2). The OsCPS1-N91 fragment was fused to the 5’ terminus of the OsCPS2-N108 fragment using an In-Fusion HD cloning kit (Takara Bio) and subcloned into the pGEM-T Easy vector. Each insert was ligated into a pTH121 vector (Zhu *et al.*, 1997) using the *Xba*I and *Bam*HI sites. Each constructed plasmid was introduced into second-leaf sheaths of 1-week-old rice seedlings using the PDS-1000 He Biolistic Particle Delivery System (Bio-Rad, CA, USA). Following bombardment, the cells were incubated on solid Murashige and Skoog (MS) medium for 24h at 28 °C in the dark before observation. Microscopic observation was performed with an Olympus AX80T microscope (Olympus, Tokyo, Japan).

### β-Glucuronidase (GUS) assay

The *GUS* cDNA and *NOS* terminator originating in the pBI221 vector were inserted into the binary vector pZH2B (Kuroda *et al.*, 2010) using *Bam*HI and *Eco*RI sites (pZH2B-GUS-Nos; Supplementary Fig. S3 at *JXB* online). The genomic DNA fragment of 2.1kb 5’ upstream of the *OsCPS1* ATG start site plus the coding sequence in the second exon of *OsCPS1* (OsCPS1p) was amplified by PCR using KOD Plus version 2 and primer set IF-AscI-CPS1p-F/IF-BamHI-CPS1p-R (Supplementary Table S3), and directly subcloned into pZH2B-GUS-Nos, which was digested with *Asc*I and *Bam*HI, using an In-Fusion HD cloning kit (Supplementary Fig. S3). The plasmid pZH2B-OsCPS1p::GUS was introduced into Nipponbare rice cells through *Agrobacterium* transfection following the method previously described (Kuroda *et al.*, 2010). Transgenic rice was grown and used for GUS assay. GUS assay was performed as previously described (Yamaguchi *et al.*, 2001), using an Axioplan 2 microscope system (Zeiss Japan, Tokyo, Japan).

### Complementation experiments

The *NOS* terminator originating in the pBI221 vector was inserted into pZH2B using *Sac*I and *Eco*RI sites (pZH2B-Nos; Supplementary Fig. S3 at *JXB* online). The full-length open reading frame (ORF) cDNA of *OsCPS2* was amplified by RT–PCR using the primer set KpnI-CPS2-F and KpnI-CPS2-R (Supplementary Table S) and subcloned into the pZH2B-Nos vector using the *Kpn*I site (Supplementary Fig. S3). The OsCPS1p fragment was cleaved from the pZH2B-OsCPS1p::GUS plasmid and ligated into pZH2B-OsCPS2 using *Asc*I and *Sma*I sites (pZH2B-OsCPS1p::OsCPS2; Supplementary Fig. S3). A heterozygous M_1_ plant of *oscps1-1* was selected as a transformation host by PCR genotyping using MightyAmp DNA polymerase (Takara Bio) and the primer sets CPS1-WT-F/CPS1-WT-R (~230bp) for the wild-type gene and Tos17-F/CPS1-WT-R (~500bp) for the mutant gene (Supplementary Fig. S1). pZH2B-OsCPS1p::OsCPS2 was introduced into the heterozygous rice plant by *Agrobacterium* infection (Kuroda *et al.*, 2010), and T_1_ seeds of 12 transgenic lines were obtained. The T_1_ seedlings were grown and subjected to genotyping. The host *OsCPS1* genotype was confirmed by PCR using the above primer sets, and the transgene *OsCPS2* cDNA was detected by PCR using the primer set CPS2-QRT-F/CPS2-QRT-R (Supplementary Table S1), ~250bp from the endogenous gene (with intron) and ~170bp from the transgene cDNA (no intron). The targeted-genotype plants (homozygous *oscps1-1* mutants with *OsCPS2* cDNA and segregated wild-type plants without *OsCPS2* cDNA) were grown and T_2_ seeds were obtained for further analyses. The upper and basal parts of T_2_ seedlings were collected and used for gene expression analysis, after genotyping of each seedling by PCR, as described above.

## Results

### Subcellular localization of OsCPS1 and OsCPS2

**Fig. 2.**
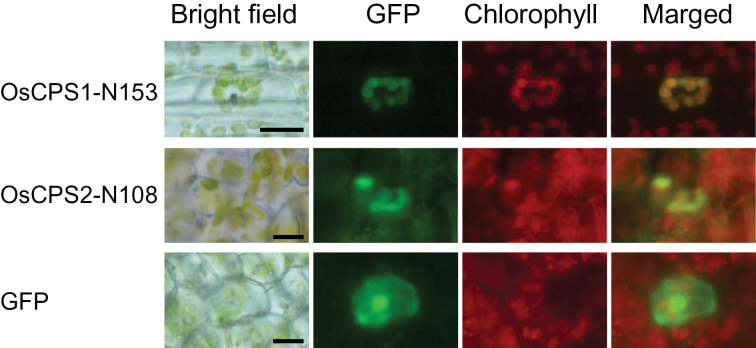
Subcellular localization of OsCPS1 and OsCPS2. The N-terminal region including the transit peptide of each CPS fused to GFP at its N-terminus was expressed in rice mesophyll cells, and GFP fluorescence was observed by fluorescence microscopy, as described in the Materials and methods. OsCPS1-N153 and OsCPS2-N108 indicate the N-terminal 153 and 108 amino acids of OsCPS1 and OsCPS2, respectively. Scale bars indicate 10 μm.

Diterpene cyclases, including *ent-*CDP synthase and *ent*-kaurene synthase, in higher plants have been considered to localize in the plastid (Toyomasu and Sassa, 2010). In general, transit peptides for plastid targeting are present at the N-termini of the plant diterpene cyclases (Toyomasu and Sassa, 2010), and *Arabidopsis* CPS, which is responsible for GA biosynthesis, has been shown to be transported into the plastid (Sun and Kamiya, 1994). OsCPS1 (867 amino acids, LOC_Os02g17780) and OsCPS2 (800 amino acids, LOC_Os02g36210) also have transit peptide-like sequences at their N-termini (Otomo *et al.*, 2004; Prisic *et al.*, 2004). To verify the plastid localization of OsCPS1 and OsCPS2, the N-terminal transit peptide-like sequences OsCPS1-N153 and OsCPS2-N108 (Supplementary Fig. S2A at *JXB* online) were fused to GFP and expressed in rice mesophyll cells. GFP fluorescence in plastids indicated that both OsCPS1-N153–GFP and OsCPS2-N108–GFP were transported into the plastids (Fig. 2). These results suggest that OsCPS2 as well as GA biosynthetic OsCPS1 localize in the rice cell plastid.

### Expression patterns of *OsCPS1* and *OsCPS2* in the second-leaf sheath of rice seedlings

**Fig. 3.**
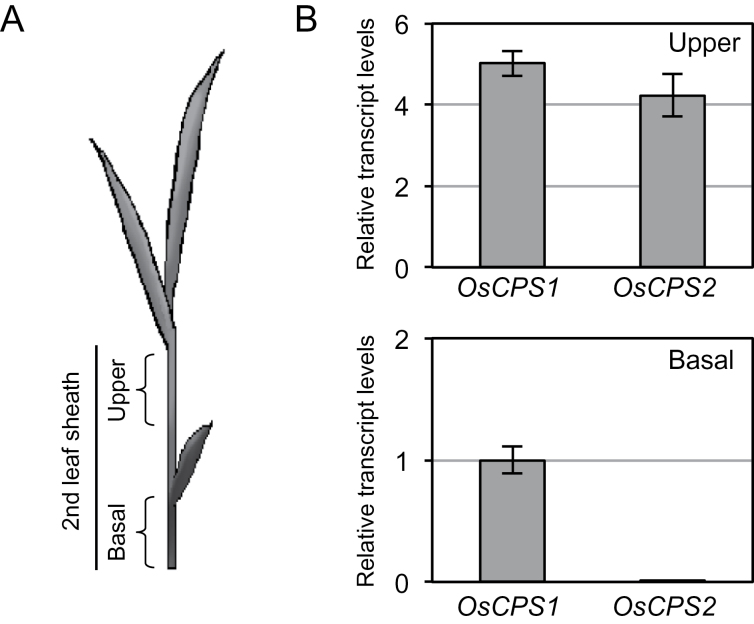
Localization of transcripts of *OsCPS1* and *OsCPS2* in the second-leaf sheath of rice. (A) The upper and basal parts of the rice second-leaf sheath were used for expression analyses by qRT–PCR. (B) Relative transcript levels of two CPS genes. The concentration of each transcript was normalized to that of 18S rRNA, and adjusted to a value of 1 for *OsCPS1* in the basal parts with SEs (*n*=3).

The data suggested the same subcellular localization of OsCPS1 and OsCPS2 in the rice cell. Next, expression analysis of *OsCPS1* and *OsCPS2* was performed to compare the localization of these transcripts in rice plants. The second-leaf sheath grows sensitively in response to exogenously applied GA; therefore, it has been used in bioassays to assess GA activity (Murakami, 1968; Nishijima and Katsura, 1989). The second-leaf sheath was used here as material for qRT–PCR analysis. The upper and basal 2cm were excised from second-leaf sheaths of third-leaf stage rice seedlings (Fig. 3A) and used for RNA extraction. The qRT–PCR analysis showed that the *OsCPS2* transcript level was much lower than that of *OsCPS1* in the basal part including the meristem tissues, whereas *OsCPS2* expression was almost similar to *OsCPS1* expression in the upper part (Fig. 3B).

**Fig. 4.**
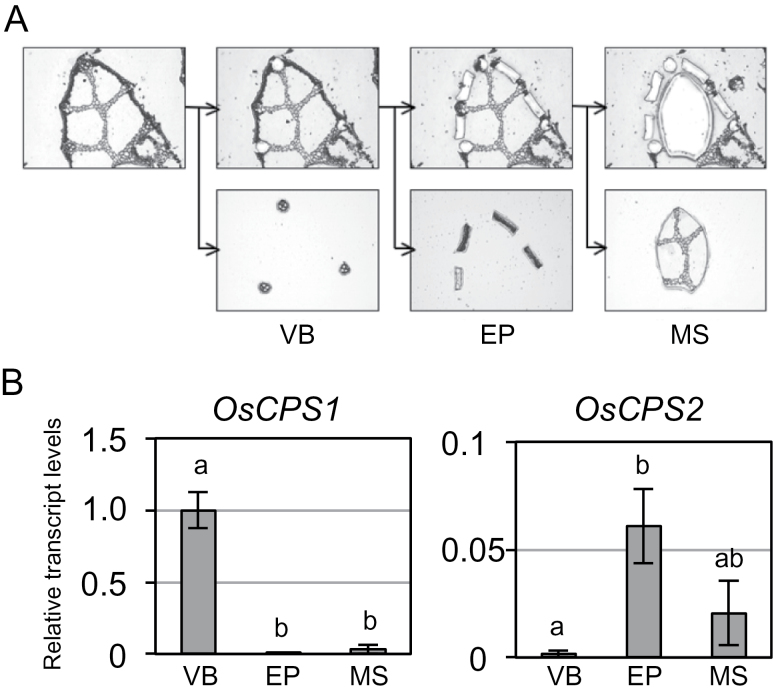
Localization of transcripts of *OsCPS1* and *OsCPS2* to different tissues in the upper part of the second-leaf sheath of rice. (A) Tissue samples collected by laser microdissection. Vascular bundle-rich (VB), epidermal-rich (EP), and mesophyll-rich (MS) tissues were collected from segments of the upper parts of the second-leaf sheaths of rice. (B) Relative transcript levels of two rice CPS genes in VB, EP, and MS tissues. Expression analysis was performed by qRT–PCR. The concentration of each transcript was normalized to that of 18S rRNA and adjusted to a value of 1 for *OsCPS1* in VB with SEs (*n*=3). Data were subjected to analysis of variance (ANOVA) in each graph.

Furthermore, the transcript contents were quantified in separate tissues from the upper part of the second-leaf sheath in which the transcript contents of the two *OsCPS* genes were almost similar. Vascular bundle-rich, epidermis-rich, and residual tissues (mesophyll-rich tissues) were excised from leaf sheath slices, collected by laser microdissection (Fig. 4A), and used for RNA extraction. qRT–PCR was performed using amplified cDNAs derived from RNA obtained as a template. *OsCPS1* transcripts were significantly more abundant in vascular bundle-rich tissues than in other tissues, whereas *OsCPS2* transcript levels were more abundant in epidermis-rich and mesophyll-rich tissues than in vascular bundle-rich tissues (Fig. 4B). Expression of the GA biosynthetic gene *AtCPS*, which encodes *ent*-CDP synthase in *Arabidopsis*, is observed around vascular tissues (Silverstone *et al.*, 1997; Yamaguchi *et al.*, 2001), similar to *OsCPS1*, but unlike *OsCPS2*.

### Complementation of the severe dwarf phenotype of the *OsCPS1* mutant by *OsCPS2* expression under the *OsCPS1* promoter

**Fig. 5.**
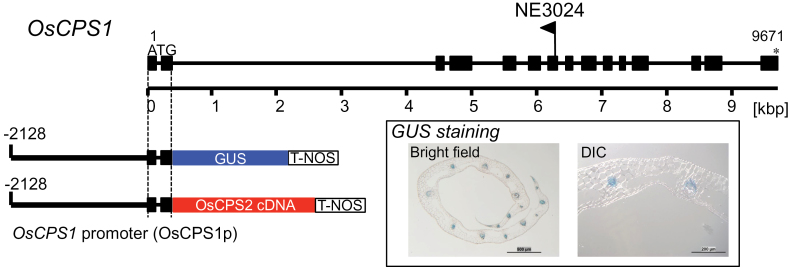
Gene structure of *OsCPS1* and its promoter region used in the complementation experiment. Filled boxes represent exons and lines represent introns of *OsCPS1* from the translation start codon to the stop codon. The flag shows the insertion site of *Tos17* in the *oscps1-1* mutant (NE3024). The 2.1kb region of the *OsCPS1* promoter (OsCPS1p) used for GUS assay and ectopic expression of *OsCPS2* is shown. The inset shows images of the sliced second-leaf sheath of GUS-stained rice T_1_ seedlings into which OsCPS1p::*GUS* was introduced. Bars in the left panel (bright field) and right panel (differential interference contrast microscopy, DIC) indicate 500 μm and 200 μm, respectively. (This figure is available in colour at *JXB* online.)

A complementation experiment with a loss-of-function *OsCPS1* mutant by *OsCPS2* under the regulation of the *OsCPS1* promoter was performed to confirm genetically that the *OsCPS1* and *OsCPS2* expression sites are different in rice. First a suitable DNA region of the *OsCPS1* promoter wase identified using a *GUS* reporter gene. A genomic DNA fragment of 2.1kb 5′ upstream of the *OsCPS1* ATG start site plus the coding sequence in the second exon of *OsCPS1* (OsCPS1p) was amplified using PCR and fused to *GUS* (Fig. 5) in the pZH2B binary vector (Kuroda *et al.*, 2010). It has been shown previously that the first 1–2 introns of *AtCPS* are required for proper expression in *Arabidopsis* (Silverstone *et al.*, 1997). GUS staining was observed in the vascular bundle of the second-leaf sheath of rice seedlings (Fig. 5; Supplementary Fig. S4 at *JXB* online). The close correspondence of GUS staining to the accumulation pattern of *OsCPS1* transcripts by tissue-specific qRT–PCR (Fig. 4B) suggested that OsCPS1p promoter activity was effective. When the OsCPS1p fragment is fused to the full-length *OsCPS2* ORF cDNA in-frame, the resulting translated product is OsCPS2, which has the OsCPS1-N91 at its N-terminus (Supplementary Fig. S2B). Both OsCPS1-N91 and a chimeric peptide OsCPS1-N91:OsCPS2-N108 led GFP to the plastid (Supplementary Fig. S5), similar to OsCPS2-N108 (Fig. 2). These results suggest that a pseudo-mature form of OsCPS2 is transported into plastids when driven by OsCPS1p.

**Fig. 6.**
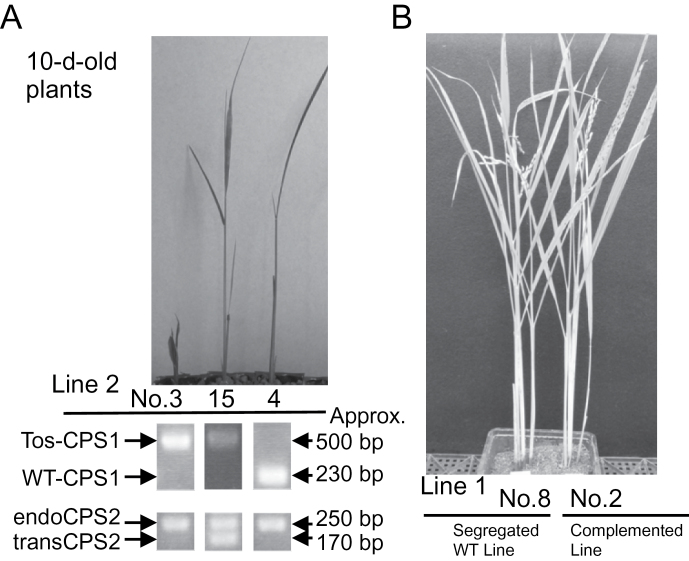
Complementation experiment of a loss-of-function *OsCPS1* mutant by ectopic expression of *OsCPS2*. T_1_ seeds were obtained from several lines of T_0_ plants, transgenic heterozygous *oscps1-1* (NE3024) plants into which OsCPS1p::*OsCPS2* cDNA (Fig. 5) was introduced. (A) Results of genotyping and images of T_1_ seedlings of line 2, the knockout line (no. 3), complemented line (no. 15), and the segregated wild-type line (no. 4). Tos-CPS1, bands from *Tos17*-inserted *OsCPS1* genome DNA (Tos17-F and CPS1-WT-R in Supplementary Fig. S1 available at *JXB* online); WT-CPS1, bands from wild-type *OsCPS1* genomic DNA (CPS1-WT-F and CPS1-WT-R in Supplementary Fig. S1); endoCPS2, bands from native *OsCPS2* genomic DNA (with intron); transCPS2, bands from transgene *OsCPS2* cDNA (no intron). The sense primer (CPS2-QPCR-F; Supplementary Table S) and antisense primer (CPS2-QPCR-R; Supplementary Table S1) for *OsCPS2* genotyping were designed from the nucleotide sequences of exons 10 and 11, respectively. (B) Images of adult T_1_ rice plants of line 1, the segregated wild-type line (no. 8), and the complemented line (no. 2).

**Fig. 7.**
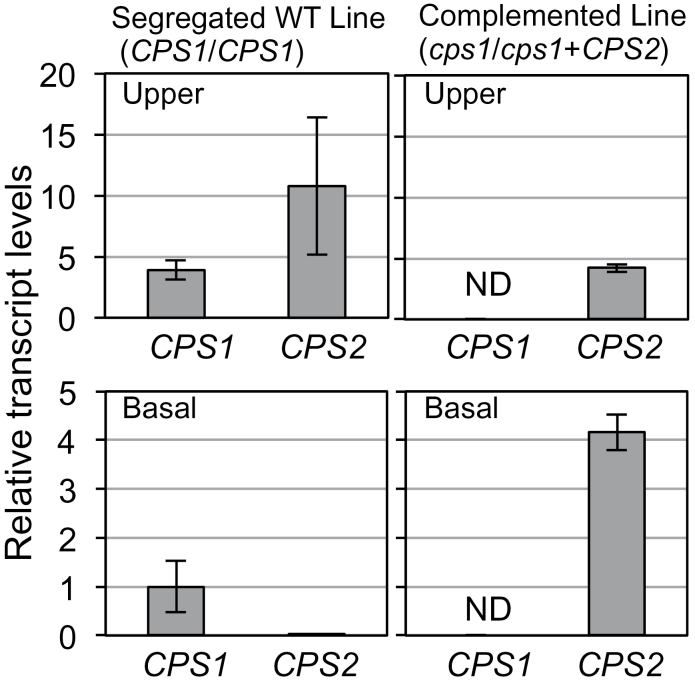
Expression patterns of *OsCPS1* and *OsCPS2* in second-leaf sheaths of the complemented line rice seedlings. The upper and basal parts of the second-leaf sheath (Fig. 3A) of T_2_ seedlings, obtained from the segregated wild-type T_1_ line (line 1, no. 8) and the complemented T_1_ line (line 1, no. 2) rice plants, were used for expression analyses by qRT–PCR. The concentration of each transcript was normalized to that of 18S rRNA, and adjusted to a value of 1 for *OsCPS1* in the basal parts of the segregated wild-type line plants with SEs (*n*=3). ND, not detected.

The OsCPS1p fragment was accordingly fused to *OsCPS2* ORF cDNA (Fig. 5) in the pZH2B vector and introduced into a heterozygous *oscps1-1* mutant by *Agrobacterium* infection. *oscps1-1* is a *Tos17*-inserted *OsCPS1* mutant that displays a severe dwarf phenotype caused by GA deficiency (Sakamoto *et al.*, 2004). Because the homozygous *oscps1-1* mutant was incapable of dedifferentiation, a heterozygous mutant was transformed by *Agrobacterium* infection, and self-pollination of transgenic plants produced T_1_ seeds. Transgenic homozygous *oscps1-1* (complemented line), non-transgenic homozygous *oscps1-1* (knockout line), and non-transgenic wild-type (segregated wild-type line) plants were observed by genotyping the T_1_ seedlings (Fig. 6A). Four complemented line T_1_ plants were identified, and displayed almost the same height as that of the segregated wild-type lines (Fig. 6B). *OsCPS1* and *OsCPS2* expression patterns were analysed in the second-leaf sheath to check successful ectopic expression of *OsCPS2*. qRT–PCR showed that *OsCPS2* transcripts accumulated in basal parts of the second-leaf sheath of T_2_ complemented line seedlings, similar to *OsCPS1* transcripts in wild-type Nipponbare (Fig. 3B) and in segregated wild-type line seedlings, whereas wild-type *OsCPS1* transcripts were not detected in complemented line plants (Fig. 7). These results indicate that *OsCPS2* expression under the *OsCPS1* promoter rescued the *oscps1-1* mutant phenotype.

## Discussion

Phytohormone GAs are biosynthesized from GGDP, a common precursor of diterpenoids, through several steps catalysed by various biosynthetic enzymes including diterpene cyclases in plastids, P450 monooxygenases in the endoplasmic reticulum, and 2-oxoglutarate-dependent dioxygenases in the cytoplasm (Yamaguchi, 2008; Hedden and Thomas, 2012). The sites of expression of genes encoding the soluble dioxygenases GA 20-oxidase and GA 3-oxidase, which are responsible for direct synthesis of physiologically active GAs, have been characterized in rice previously (Kaneko *et al.*, 2003), whereas the sites of expression of diterpene cyclase genes, including *OsCPS1* and *OsKS1*, involved in the initial step of bioactive GA biosynthesis have not been elucidated. The diterpene cyclases responsible for biosynthesis of *ent*-kaurene, a GA biosynthetic intermediate, have been identified not only in plants but also in bacteria (Morrone *et al.*, 2009) and fungi (Kawaide *et al.*, 1997; Toyomasu *et al.*, 2000), both of which produce GAs as specialized metabolites. Although two distinct cyclases successively convert GGDP into *ent*-kaurene via *ent*-CDP in higher plants, one peptide bi-functional diterpene cyclase produces *ent*-kaurene from GGDP in bryophytes (Hayashi *et al.*, 2006; Kawaide *et al.*, 2011). Coniferous gymnosperms also have *ent*-CDP synthase and *ent*-kaurene synthase for GA biosynthesis, while they have bi-functional labdane-related diterpene cyclases involved in specialized metabolites, resin acids produced via (+)-CDP (Keeling *et al.*, 2010; Zerbe *et al.*, 2012). Synthesis of *ent*-CDP is the first branch point of GA biosynthesis from GGDP, but *ent*-CDP and *ent*-kaurene may not be specific intermediates of only GAs. For example, *Stevia rebaudiana* produces steviol glycosides via *ent*-kaurene. In *S. rebaudiana*, *ent*-kaurene is synthesized by highly accumulated *ent*-CDP synthase and *ent*-kaurene synthase, both of which are responsible for GA biosynthesis, in leaf parenchyma (Richman *et al.*, 1999). In addition, rice produces labdane-related phytoalexins converted from *ent*-CDP; phytocassanes A–E and oryzalexins A–F. Rice has an *ent*-CDP synthase, OsCPS2, specific for phytoalexin biosynthesis as well as OsCPS1 specific for GA biosynthesis (Otomo *et al.*, 2004; Prisic *et al.*, 2004), whereas *S. rebaudiana* uses *ent*-CDP synthase for GA biosynthesis to synthesize steviol glycosides.

Here it is shown that transcript levels of *OsCPS2* were drastically lower than those of *OsCPS1* in the basal parts of rice seedling second-leaf sheath. The basal parts include the meristematic tissues necessary for rice growth. It has been shown that growth signals mediated by GAs engage in antagonistic cross-talk with defence signals mediated by jasmonic acid via the DELLA–JAZ interaction (Hou *et al.*, 2013). DELLA and JAZ are key repressors in GA and jasmonic acid signalling, respectively. Therefore, expression of the defence gene *OsCPS2* might be suppressed in the basal parts with high growth activity, although it has been unclear whether cross-talk of these hormones is involved in the *OsCPS2* regulation in the basal parts of rice seedlings. Furthermore, qRT–PCR analysis using separate tissues prepared by laser microdissection indicated that *OsCPS1* transcripts mainly accumulated in vascular bundle-rich tissues, whereas *OsCPS2* transcripts were mainly observed in epidermis-rich tissues. Thus, the different localization of *OsCPS1* and *OsCPS2* transcripts in rice plants was found.On the other hand, it was suggested that both OsCPS2 and OsCPS1 function in the plastid. It was previously shown that GAs are biosynthesized mainly from GGDP that is derived through the methylerythritol 4-phosphate (MEP) pathway in the plastid (Kasahara *et al.*, 2002). Transcript levels of several genes in rice responsible for the MEP pathway are drastically up-regulated after elicitor treatment, suggesting that the MEP pathway also participates in diterpene phytoalexin biosynthesis in rice (Okada *et al.*, 2007). Therefore, it is reasonable that OsCPS2 functions in the plastid like GA biosynthetic OsCPS1.

Although recombinant OsCPS2 converts GGDP to *ent*-CDP *in vitro*, *OsCPS2* cannot rescue loss-of-function *OsCPS1* mutants under control of the native promoter (Sakamoto *et al.*, 2004). In the present study, it was shown that *OsCPS2* under regulation by the *OsCPS1* promoter complemented the *OsCPS1* mutant phenotype. The qRT–PCR results showed differences in tissue-specific expression of the two CPS genes. These results strongly suggest that proper tissue-specific expression of *ent*-CDP synthase genes is critical for GA biosynthesis. Results of a *GUS* reporter gene assay in germinating *Arabidopsis* seeds suggest that *AtCPS* transcripts for GA biosynthesis localize in vascular tissues, although no signal was detected by *in situ* hybridization (Silverstone *et al.*, 1997). Another study showed that transcripts of dioxygenases, responsible for later steps of GA biosynthesis, localize mainly in the cortex and endodermis, and promoter-swapping experiments indicate that intercellular transport of the biosynthetic intermediate *ent*-kaurene is required to produce bioactive GAs (Yamaguchi *et al.*, 2001). An *SrCPS* signal involved in stevioside biosynthesis in *S. rebaudiana* was detected in mesophyll by *in situ* hybridization, whereas its signal for GA biosynthesis in vascular tissues was not detected using this method (Richman *et al.*, 1999), similar to *AtCPS* (Silverstone *et al.*, 1997). It has been suggested that a spatially different localization of *CPS* transcripts separates stevioside biosynthesis from GA biosynthesis in *S. rebaudiana*. In any case, *CPS* expression patterns associated with GA biosynthesis are considered well conserved among higher plant species. However, it is reasonable that transcription of stress-inducible *OsCPS2* mainly occurs near the epidermis, producing phytoalexins in response to environmental stress. The present results confirm that the first step in GA biosynthesis occurs in the vascular tissues for effective production of bioactive GAs. The monocots wheat and maize not only have the *ent*-CDP synthase genes *TaCPS3* and *An1*, which are responsible for GA biosynthesis, but also have stress-inducible *ent*-CDP synthase genes *TaCPS1* and *An2* (Harris *et al.*, 2007; Wu *et al.*, 2012). Similar to rice, *TaCPS1* and *An2* may be expressed in different tissues from those of the GA biosynthetic genes *TaCPS3* and *An1*.

It was shown herein that *OsCPS1* and *OsCPS2* transcripts were differentially localized in rice plants according to their distinct biological roles, one for growth and the other for defence, although these translated products have the same enzymatic activity *in vitro*. It is concluded that *OsCPS2* contributes little to GA biosynthesis and cannot prevent the GA-deficient dwarf phenotype of loss-of-function *OsCPS1* mutants because of different localization of *OsCPS2* transcripts from *OsCPS1* transcripts.

## Supplementary data

Supplementary data are available at *JXB* online.

Figure S1. Primer design for transcript analyses and genotyping

Figure S2. Transit peptide-like sequences of OsCPSs

Figure S3. Construction of plasmids for introducing the transgene

Figure S4. GUS staining of rice seedlings.

Figure S5. Subcellular localization of GFP fused to OsCPS1-N91 and OsCPS1-N91:OsCPS2-N108 at its N-terminus

Table S1. Sequences of primers used for qRT–PCR.

Table S2. Sequences of primers used for GFP experiments.

Table S3. Sequences of primers used for complementation experiments.

Supplementary Data

## Abbreviations:

CDP: copalyl diphosphate
GA: gibberellin
GFP: green fluorescent protein
GGDP: geranylgeranyl diphosphate
GUS: β-glucuronidase
MEP: methylerythritol 4-phosphate
qRT–PCR: quantitative reverse transcription–PCR.
