# Supplementary material for: Transcripts of two ent-copalyl diphosphate synthase genes differentially localize in rice plants according to their distinct biological roles
Source: J Exp Bot. 2014 Oct 21;66(1):369–76. doi: 10.1093/jxb/eru424 (PMC4265168; doi:10.1093/jxb/eru424)
Supplement: Supplementary Data [file supp_66_1_369__index.html]

Transcripts of two ent-copalyl diphosphate synthase genes differentially localize in rice plants according to their distinct biological roles — Transcripts of two ent-copalyl diphosphate synthase genes differentially localize in rice plants according to their distinct biological roles — Supplementary Data 

# Transcripts of two *ent*-copalyl diphosphate synthase genes differentially localize in rice plants according to their distinct biological roles

## Supplementary Data

Data files

**Files in this Data Supplement:**

- Supplementary Data - Supplementary Data
