# Supplementary material for: Transcripts of two ent-copalyl diphosphate synthase genes differentially localize in rice plants according to their distinct biological roles
Source: J Exp Bot. 2014 Oct 21;66(1):369–76. doi: 10.1093/jxb/eru424 (PMC4265168; doi:10.1093/jxb/eru424)
Supplement: Supplementary Data [file supp_eru424_jexbot130641_file001.pdf]

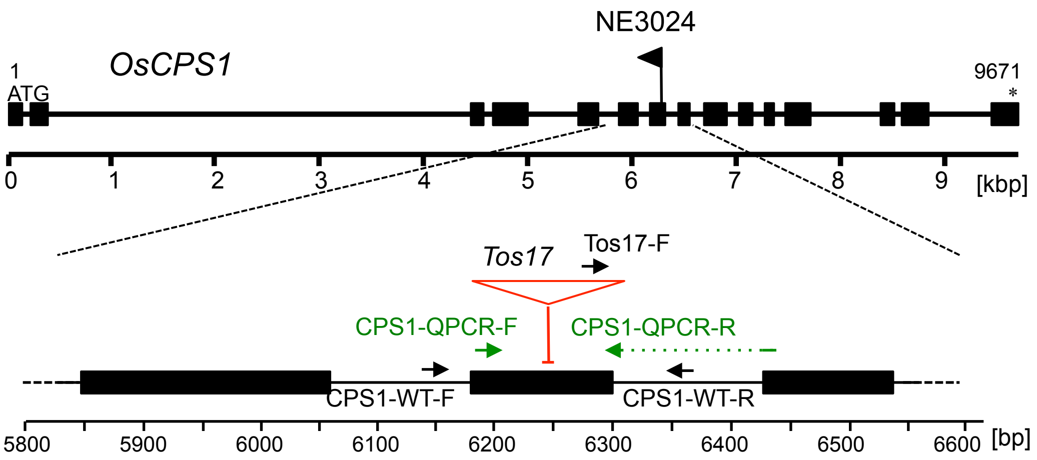

Supplementary Fig. S1. Primer design for transcript analyses and genotyping of *OsCPS1*. Schematic drawing of insertion site of retrotransposon *Tos17* in *oscps1-1* mutant (NE3024) is expanded. Green and black arrows indicate primer sets used for transcript analyses (CPS1-QRT-F and CPS1-QRT-R; Supplemental Table S1) and genotyping (Tos17-F, CPS1-WT-F and CPS1-WT-R; Supplemental Table S3), respectively.

A

```
OsCPS1 --MIHLHSPPTAPAAFGGAGSADWRRRRRWSWSSSSRAPVAKGGHLRPCVWRRGGDDGGG  58
OsCPS2 MQMQVLTAASSLPRATLLRPAAAEPWRQSFLQLQAR--PIQRPGIMLHCKAQLQGQE---  55
         *  * :..: * *     :*    *: :   .:   *: : * :  *  :  *::

OsCPS1 EDHHADGGGGGGGGAAWRARATTAGVSSSSSTAKGLQANIIEHETPRITKWPNESRDLDD 118
OsCPS2 -------------------------------TRERRQLDDDEHARP--------------  70
                                      * :  * :  **  *

OsCPS1 HQQNNEADEEADDELQPLVEQVRSMLSSMED--GAITASAYDTAWVALVPRLDGEG--GT 174
OsCPS2 PQGGDDDVAASTSELPYMIESIKSKLRAARNSLGETTVSAYDTAWIALVNRLDGGGERSP 130
        * .::    : .**  ::*.::* * : .:  *  *.*******:*** **** *  ..

OsCPS1 QFPAAVRWIVGSQLADGSWGDEALFSAYDRVINTLACVVALTRWSLHHDQCKQGLQFLNL 234
OsCPS2 QFPEAIDWIARNQLPDGSWGDAGMFIVQDRLINTLGCVVALATWGVHEEQRARGLAYIQD 190
       *** *: **. .**.****** .:* . **:****.*****: *.:*.:*  :** :::
```

B

OsCPS1-N91:OsCPS2-N108

MIHLHSPPTAPAAFGGAGSADWRRRRRWSWSSSSRAPVAKGGHLRPCVWRRGGDDGGGEDHHAD
GGGGGGGGAAWRARATTAGVSSSSSTAgspgtMQMQVLTAASSLPRATLLRPAAAEPWRQSFLQ
LQARPIQRPGIMLHCKAQLQGQETRERRQLDDDEHARPPQGGDDDVAASTSELPYMIESIKSKL
RAARNSLGETTV

Supplementary Fig. S2. Transit peptide-like sequences of OsCPSs. A, Deduced amino acid sequences of N-termini of OsCPSs. Green and red characters indicate the N-terminal 153 amino acids of OsCPS1 (OsCPS1-N153) and the N-terminal 108 amino acids of OsCPS2 (OsCPS2-N108), respectively, both of which were used in GFP experiments (Fig. 2). Green boldface characters inidicate the N-terminal 91 amino acids of OsCPS1 (OsCPS1-N91) encoded by the first and second exons of OsCPS1p (Fig. 5). B, Deduced amino acid sequences of chimeric transit peptide-like translated product derived from pZH2B-OsCPS1p::OsCPS2 (Fig. 5). Lowercase black characters between OsCPS1-N91 and OsCPS2-N108 show the deduced five amino acids encoded by the residual multi-cloning-site fragment (Supplemental Fig. S3).

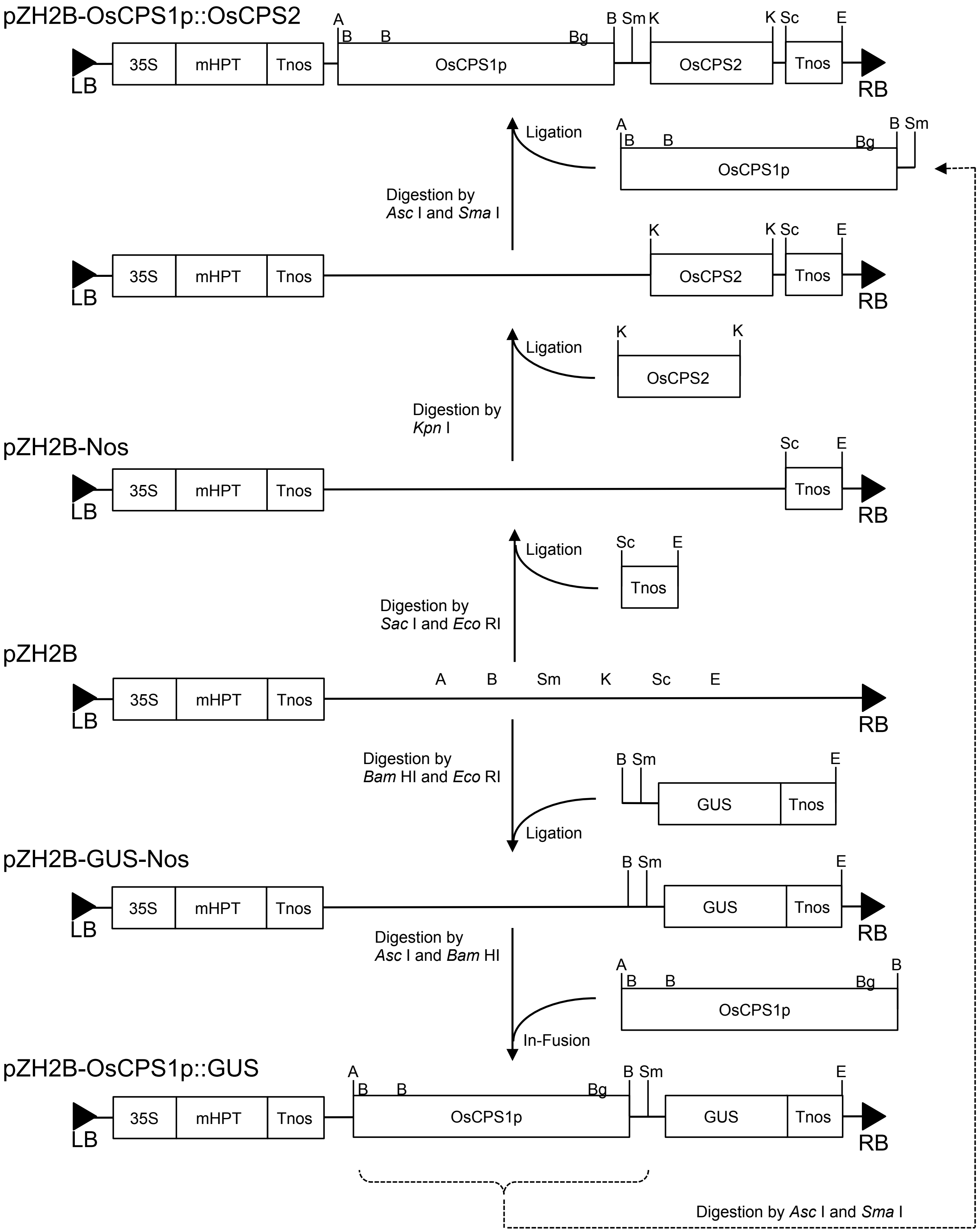

Supplementary Fig. S3. Construction of plasmids for introducing transgene. Restriction endonuclease sites: A, *Asc*I; B, *Bam*HI; Bg, *Bgl*II; E, *Eco*RI; K, *Kpn*I; Sc, *Sac*I; Sm, *Sma*I. Antibiotic gene: mHPT. Other DNA fragments: 35S, CaMV35S promoter; Tnos, NOS terminator; LB, T-DNA left border, RB, T-DNA right border. OsCPS1p has two *Bam*HI sites and one *Bgl*II site. The In-Fusion reaction was performed with an In-Fusion HD cloning kit (Takara Bio), as described in the Materials and methods section.

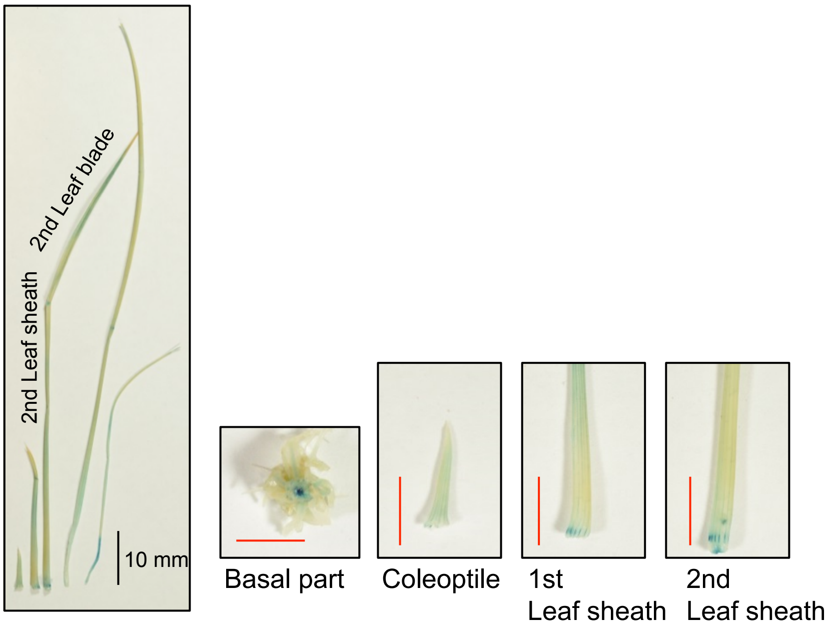

Supplementary Fig. S4. GUS staining of a four-leaf-stage rice seedling. Images show a T1 line plant into which pZH2B-OsCPS1p::GUS was introduced. The OsCPS1p::*GUS* construct and GUS staining of the sliced second-leaf sheath are shown in Fig. 5. Linear GUS staining was observed in vascular-bundle tissues in the leaf sheath. Black and red bars indicate 10 mm and 5 mm, respectively.

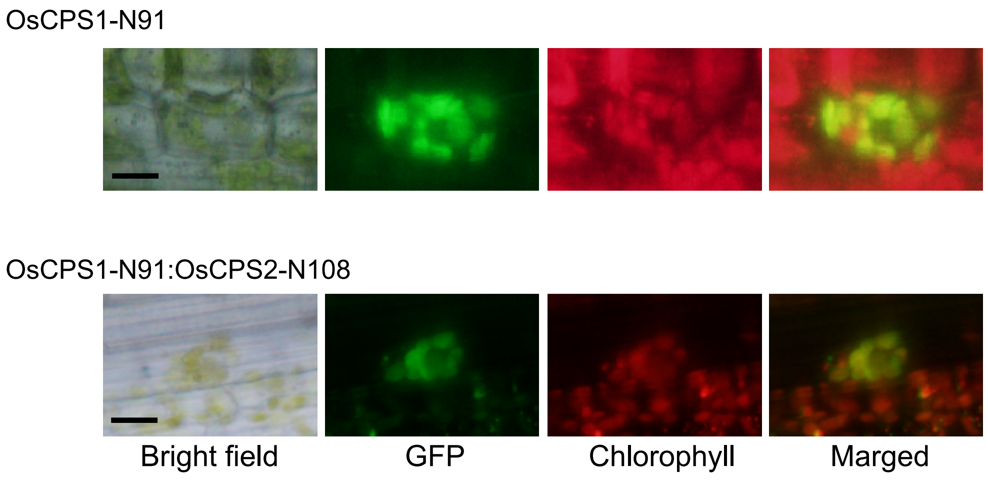

Supplementary Fig. S5. Subcellular localization of GFP fused to the N-terminal 91 amino acids of OsCPS1 (OsCPS1-N91) and the chimeric peptide OsCPS1-N91:OsCPS2-N108 at its N terminus. OsCPS1-N91:OsCPS2-N108 is shown in Supplementary Fig. S2B. GFP fluorescence was observed under fluorescence microscopy, as described in Materials and methods. Bars indicate 10 μm. GFP control is shown in Fig. 2.

Supplemental Table S1. Sequences of primers used for qRT-PCR

Name	Sequence (5' -> 3')
CPS1-QPCR-F	GAACGTTTACCCGGTCGATC
CPS1-QPCR-R	CTTCAGTCCAGTGCCTGTTG
CPS2-QPCR-F	CGAGGAGCTTACTGTACGC
CPS2-QPCR-R	TGAGCAGATCTCGATTGTG
18S-rRNA-QRT-F	GGAGCGATTTGTCTGGTTA
18S-rRNA-QRT-R	ATCTAAGGGCATCACAGACC

CPS2-QPCR-F and CPS2-QPCR-R were used not only for qRT-PCR but also for genotyping.

Supplemental Table S2. Sequences of primers used for GFP experiments

Name	Sequence (5’ -> 3’)
XbaI-CPS1-F	tctagaATGATTCACCTCCACTCCCCGCCGACGGCG
BamHI-CPS1-N153-R	ggatccGGTGATCGCGCCGTCCTCCAT
XbaI-CPS2-F	tctagaATGCAGATGCAGGTGCTCAC
BamHI-CPS2-N108-R	ggatccGACGGTGGTCTCGCCGAGG
CPS1N91CPS2-F	GCCGGATCACCGGGTACCatgcagatgcaggtgctcac
CPS1N91CPS2-R	catGGTACCCGGTGATCCGGCTGTACTGCTGGAGCTCG

Small captures in XbaI-CPS1-F, XbaI-CPS2-F and XbaI-CPS4-F show *Xba*I site. Small captures in BamHI-CPS1-N153-R, BamHI-CPS2-N108-R and BamHI-CPS4-N101-R show *Bam*HI site. Small captures and underlined letters in CPS1N91CPS2-F and CPS1N91CPS2-R show sequences of 5’ end of OsCPS2 ORF and sequences of residual multi-cloning-site fragment between OsCPS1p and OsCPS2 in pZH2B (Supplemental Fig. S2B and S3). Double-underlined characters represent substituted nucleotides for the deletion of the *Bam*HI site in the residual multi-cloning-site fragment, without changing an encoded amino acid (CPS1N91CPS2-F, C to A; CPS1N91CPS2-R, G to T). The overlap region of CPS1N91CPS2-F and CPS1N91CPS2-R, including the residual multi-cloning-site sequences, is for the In-Fusion reaction, as described in the Materials and methods section.

Supplemental Table S3. Sequences of primers used for complementation experiments

Name	Sequence (5’ -> 3’)
IF-AscI-CPS1p-F	atgttactaggcgcgCCTACGCCATATCATTGCCTTTATC
IF-BamHI-CPS1p-R	gaccacccggggatcCGGCTGTACTGCTGGAGCTCGACAC
KpnI-CPS2-F	ggtaccATGCAGATGCAGGTGCTCA
KpnI-CPS2-R	ggtaccTAATTGACATCCTCGAACA
Tos17-F	TGACAACACCGGAGCTATAC
CPS1-WT-F	GCAGCTGATCTCAGATCATG
CPS1-WT-R	ACAGAAGACACCACACATATCAG

Small captures in IF-ASCI-CPS1p-F and IF-BamHI-CPS1p-R show terminal sequences of digested pZH2B-GUS-Nos vector (Supplementary Fig. S3) for introduction by In-Fusion reaction, as described in the Materials and methods section. Small captures in KpnI-CPS2-F and KpnI-CPS2-R show *Kpn* I site. Tos17-F, CPS1-WT-F, and CPS1-WT-R are for genotyping.
